# Intestinal inflammation increases convulsant activity and reduces antiepileptic drug efficacy in a mouse model of epilepsy

**DOI:** 10.1038/s41598-019-50542-0

**Published:** 2019-09-27

**Authors:** Carmen De Caro, Antonio Leo, Valentina Nesci, Carla Ghelardini, Lorenzo di Cesare Mannelli, Pasquale Striano, Carmen Avagliano, Antonio Calignano, Paolo Mainardi, Andrew Constanti, Rita Citraro, Giovambattista De Sarro, Emilio Russo

**Affiliations:** 1 Chair of Pharmacology, Department of Health Sciences, School of Medicine, University of Catanzaro, Catanzaro, Italy; 2 Department of Neuroscience, Psychology, Drug Research and Child Health-Neurofarba-Pharmacology and Toxicology Section, University of Florence, Florence, Italy; 3 Pediatric Neurology and Muscular Diseases Unit, Department of Neurosciences, Rehabilitation, Ophthalmology, Genetics, Maternal and Child Health, University of Genoa, “G. Gaslini” Institute, Genoa, Italy; 4 Department of Pharmacy, University of Naples Federico II, Naples, Italy; 5 Medical Science Liason (MSL) Kolfarma Srl, Genoa, Italy; 6 Department of Pharmacology, UCL School of Pharmacy, 29/39 Brunswick Square, London, United Kingdom

**Keywords:** Epilepsy, Epilepsy

## Abstract

We studied the effects of intestinal inflammation on pentylenetetrazole (PTZ)-induced seizures in mice and the effects thereon of some antiepileptic and anti-inflammatory treatments to establish if a link may exist. The agents tested were: alpha-lactoalbumin (ALAC), a whey protein rich in tryptophan, effective in some animal models of epilepsy and on colon/intestine inflammation, valproic acid (VPA), an effective antiepileptic drug in this seizure model, mesalazine (MSZ) an effective aminosalicylate anti-inflammatory treatment against ulcerative colitis and sodium butyrate (NaB), a short chain fatty acid (SCFA) normally produced in the intestine by gut microbiota, important in maintaining gut health and reducing gut inflammation and oxidative stress. Intestinal inflammation was induced by dextran sulfate sodium (DSS) administration for 6 days. Drug treatment was started on day 3 and lasted 11 days, when seizure susceptibility to PTZ was measured along with intestinal inflammatory markers (*i.e*. NF-κB, Iκ-Bα, COX-2, iNOS), histological damage, disease activity index (DAI) and SCFA concentration in stools. DSS-induced colitis increased seizure susceptibility and while all treatments were able to reduce intestinal inflammation, only ALAC and NaB exhibited significant antiepileptic properties in mice with induced colitis, while they were ineffective as antiepileptics at the same doses in control mice without colitis. Interestingly, in DSS-treated mice, VPA lost part of its antiepileptic efficacy in comparison to preventing seizures in non-DSS-treated mice while MSZ remained ineffective in both groups. Our study demonstrates that reducing intestinal inflammation through ALAC or NaB administration has specific anticonvulsant effects in PTZ-treated mice. Furthermore, it appears that intestinal inflammation may reduce the antiepileptic effects of VPA, although we confirm that it decreases seizure threshold in this group. Therefore, we suggest that intestinal inflammation may represent a valid antiepileptic target which should also be considered as a participating factor to seizure incidence in susceptible patients and also could be relevant in reducing standard antiepileptic drug efficacy.

## Introduction

Neuroinflammation is clearly linked to seizure and epilepsy pathophysiology with a bidirectional interaction, since increased brain inflammation has been associated with the occurrence of seizures and epileptogenesis and, on the other hand, seizures themselves can induce inflammatory responses in the brain^1–3^. Many studies have indicated that inflammatory mediators and pathways can be considered a suitable target for the development of novel drugs effective in the treatment of epilepsy; furthermore, mounting evidence suggests that these mediators can also be considered reliable biomarkers^4,5^. Finally, a role for neuroinflammation has also been demonstrated for epilepsy comorbidities^6^.

Less is known about the role of peripheral inflammation and whether it has a role in the pathophysiology of epilepsy^7,8^. As an example, it is known that administration of the pro-inflammatory bacterial endotoxin lipopolysaccharide (LPS) can lower seizure threshold or increase seizures in some animal epilepsy models^9–11^. However, it is known that seizures are highly associated with some systemic diseases characterized by peripheral inflammation such as systemic lupus erythematosus, Behcet’s disease (blood vessel inflammation) and Chron’s disease^12^. This latter, along with other inflammatory bowel diseases, have been suggested to be an increased risk factor for neurological complications including seizures and epilepsy^13,14^. Accordingly, it was previously reported that intestinal inflammation in animal epilepsy models lowers seizure threshold, most likely through increased circulating levels of some cytokines or other inflammatory mediators^7,15^.

Alpha-lactoalbumin (ALAC) is a whey protein rich in tryptophan; it is the major protein in human colostrum (20–25% of total protein *vs* 4% of total protein in milk) and has been described to have several physiological functions in the neonatal period^16^. ALAC has been demonstrated to be effective in some animal models of epilepsy and epileptogenesis^17,18^ and patients with epilepsy^19,20^. Furthermore, it is also known to inhibit colon/intestine inflammation and more generally to contribute to the development and maintenance of gastrointestinal physiological functions^16,21^.

We herein describe the effects of ALAC on intestinal inflammation induced by dextran sulfate sodium (DSS) administration^22^ and examine its anticonvulsant effects in the pentylenetetrazol (PTZ) mouse model^18^. Moreover, we compared the effects of ALAC with those of a valproate (VPA), an established anti-seizure drug in this animal model^23^ and with those of an effective anti-inflammatory treatment against DSS-induced colitis (mesalazine; MSZ)^24^ as well as with sodium butyrate (NaB), a short chain fatty acid (SCFA) with beneficial effects on the intestinal and other tissues, including the brain^25–28^.

## Material and Methods

### Animals

Experiments were performed in 10 week-old BALB/c AnNHsd male mice (25 ± 2 g), which were purchased from Charles River (Italy). They were housed in cages (at the most, 5 mice per cage) under stable conditions of humidity (60 ± 5%) and temperature (21 ± 2 °C), kept under a reversed light/dark (12/12 h) cycle (light on at 19:00 h). All animals had free access to water and standard chow diet VRF1 (purchased from Special Diet Service-SDS). Procedures involving animals and their care were conducted in conformity with international and national law and policies (EU Directive 2010/63/EU). The procedures reported here were approved by the Institutional Committee on the Ethics of Animal Experiments (CSV) and by the Ministero della Salute. As indicated by the animal welfare protocol, all efforts were made to minimize animal suffering and to use only the number of animals necessary to produce reliable scientific data.

### Induction of colitis and treatments

**Figure 1:**
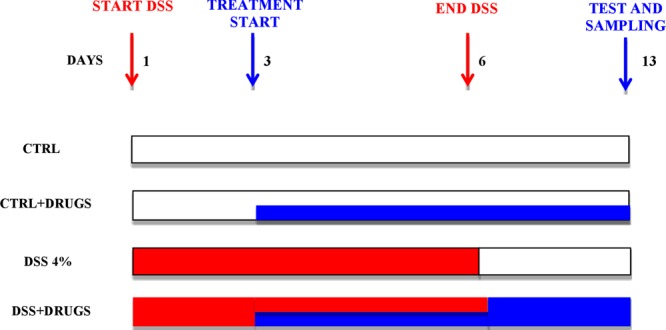
Schematic representation of experimental protocol. Drugs were given in both control (CTRL) and dextran sulfate sodium (DSS)-treated groups starting on day 3 for 10 consecutive days in the drinking water.

Experimental colitis was induced in mice by administration of 4% wt:vol DSS (36–50 kDa, MP Biomedical, Italy) in drinking water *ad libitum* from day 1 until day 6 followed by DSS-free water from day 7 until day 13 (end of experimental protocol; Fig. 1). Mice were randomly divided into the ten following groups (*n* = 40 per group) in which, when drug treatment (in drinking water^18^) was required, the drug was always given from day 3 until day 13: (1) control mice (CTR group) receiving tap water for all the experimental time; (2) mice receiving DSS from day 1 until day 6 (DSS group); (3) DSS-fed mice receiving ALAC (Kolfarma srl, Italy) at a dose of 375 mg/kg/os (DSS-ALAC); (4) DSS-fed mice receiving valproic acid (Sigma-Aldrich, Italy) at a dose of 600 mg/kg/os (DSS-VPA) (5) DSS-fed mice receiving sodium butyrate (Sigma-Aldrich, Italy) at a dose of 100 mg/kg/os (DSS-NaB) and (6) DSS-fed mice receiving mesalazine (MSZ; Sigma-Aldrich, Italy) at a dose of 15 mg/kg/os (DSS-MSZ group). The last four groups (7) ALAC (8) VPA (9) NaB and (10) MSZ received from day 3 until day 13 their respective treatments as in the DSS-fed groups. At day 13, animals were sacrificed by an i.p. injection of a mixture of ketamine/xylazine followed by cervical dislocation. For histological and western blotting analysis, only mice (at least 5 mice in each group) not receiving PTZ were used.

### Evaluation of pentylentetrazol-induced seizure susceptibility

All animals were treated with various doses of pentylentetrazol (PTZ; 30–100 mg/kg i.p.) at 15 mg intervals with at least 4 doses in each group (*n* = 7) to calculate the CD_50_ (the dose calculated inducing convulsions in 50% of animals)^29^. The doses of these drugs were injected in a volume of 0.1 ml/10 g of body weight of the mouse. The animals were then placed in isolated cages and observed 30 min after the administration of all drugs. A threshold convulsion was considered as an episode of clonic spasms lasting for at least 5 s. The absence of this threshold convulsion over 30 min indicated that the animal was protected from the convulsant-induced seizures^30^.

### Evaluation of experimental colitis and gut permeability

In all mice, their weight, presence of blood and gross stool consistency were determined daily, as previously described^22^. Each score was determined as follows:1) change in weight (0: weight loss <1% compared with the starting weight; 1: weight loss between 1 and 5%; 2: weight loss between 6 and 15%; 4: weight loss >15%); (2) stool blood (0: negative; 2: positive; 4: gross bleeding); and (3) stool consistency (0: normal; 2: loose stools; 4: diarrhea). Briefly, the disease activity index (DAI) was determined by combining the scores from these three categories and dividing that number by 3^22^.

An *in vivo* intestinal permeability assay was performed on mice using the fluorescein isothiocyanate-labeled dextran (FITC-dextran) method, as previously described^31^. Briefly, food and water were withdrawn for 6 h and mice (*n* = 5, each group) were administered by gavage with FITC-labeled dextran 4000 (60 mg/100 g body weight; Sigma-Aldrich, Milan, Italy), as a permeability tracer. After 24 hours, blood of all animals was collected by intracardiac puncture and centrifuged (3000 rpm for 15 min at RT). Then, plasma FITC-dextran concentration was determined (excitation, 485 nm; emission, 535 nm; HTS-7000 Plus-plate-reader; Perkin Elmer, Wellesley, MA, USA), using a standard curve.

### Histological analysis and scoring of colon sections

At day 13, mice were sacrificed, tissues were collected, and colon length was measured. Distal sections were stored in 10% formalin for histological analysis. Following hematoxylin and eosin (H&E) staining, colon sections were analyzed in a blinded manner by two independent observers for the evaluation of the histopathological score. The samples were given a score of 0–4 (where 0 = none; 1 = mild; 2 = moderate; 3 = severe; 4 = very severe) for epithelial injury, mononuclear infiltrate, and polymorphonuclear infiltrate^22^.

### Western blot analysis

Colons were homogenized on ice-cold lysis buffer (20 mM Tris–HCl (pH 7.5), 10 mM NaF, 150 mM NaCl, 1% Nonidet P-40, 1 mM phenylmethylsulfonyl fluoride, 1 mM Na_3_VO_4_, leupeptin and trypsin inhibitor 10 g/mL; 0.25/50 mg tissue). After 1 h, tissue lysates were obtained by centrifugation at 20,000 g for 15 min at 4 °C. Protein concentrations were estimated by the Bio-Rad protein assay using bovine serum albumin as standard.

Colon lysate proteins (70 g) were dissolved in Laemmli sample buffer, boiled for 5 min and separated on SDS-polyacrylamide gel electrophoresis and transferred to nitrocellulose membrane (240 mA for 40 min at room temperature). The filter was then blocked with 1 × phosphate buffer saline (PBS) and 3% non-fat dried milk for 40 min at room temperature and probed with anti-inducible nitric oxide synthase (iNOS) antibody (dilution 1:1000; cat. no. 610204, BD Bioscience), or anti-cyclooxygenase (COX)-2 (dilution 1:1000; cat. no. 610431, BD Bioscience, from Becton Dickinson, Buccinasco, Italy), or anti-NF-κB p65 (dilution 1:500; Santa Cruz Biotechnology, Inc., Santa Cruz, CA, United States), or anti-Iκ-Bα (dilution 1:500; Santa Cruz Biotechnology, Inc.) antibody for cytosolic and nuclear lysates, or anti-Occludin (dilution 1:500, cat. no. sc-492, Santa Cruz Biotechnology, Santa Cruz, CA, USA), in 1 × PBS, 3% non-fat dried milk, and 0.1% Tween 20 at 4 °C overnight. The secondary antibody was incubated for 1 h at room temperature. Subsequently, the blot was extensively washed with PBS, developed using enhanced chemiluminescence detection reagents (Amersham Pharmacia Biotech, Piscataway, NJ, USA) according to the manufacturer’s instructions, and the immune complex visualized by Image Quant (GE Healtcare, Milan, Italy). Nitrocellulose filter gels were repeatedly stripped for different antibodies. The protein bands were scanned and densitometrically analyzed with a model GS-700 imaging densitometer (Bio-Rad Laboratories, Milan, Italy). To ascertain that blots were loaded with equal amounts of protein lysates, they were also incubated in the presence of the antibody against the β-actin protein (dilution 1:15000, Sigma–Aldrich, Milan, Italy).

### GC-MS propionate and butyrate quantification

Fecal samples (*n* = 6 for each group) were obtained at experimental day 7 (the day after the last DSS administration) and 13 (end of all treatments), weighted (1 g) and suspended in 1 mL of saline and vortexed. After centrifugation at 4 °C, 14000 rpm for 15 min, 500 μL of supernatant was combined with 20 μL phosphoric acid (85%). Then 500 μL of ethyl acetate was added and samples were centrifugated again at 4 °C 14000 rpm for 30 min. This step was repeated three times. A quantity of the pooled extract containing the acidified propionate and butyrate were transferred into a 2-mL glass vial and loaded onto an Agilent Technologies (Santa Clara, CA, USA) 7890 gas chromatograph (GC) system with automatic loader/injector. The GC column was an Agilent DB-WAX Ultra Inert with the length 30 m, internal diameter 0.25 mm and film thickness 0.25 μm. The GC was programmed to achieve the following run parameters: the temperature of the inlet was 250 °C and the volume of injection was 1 μL. The temperature program was as follows: the initial oven temperature at 50 °C held for 1 min, ramped to 250 °C at 8 °C/min. The carrier gas was helium with a constant linear velocity of 1.5 mL/min. Pure fatty acid standards (Sigma-Aldrich) were also prepared to produce a calibration curve. Each sample was analyzed two times on the same day.

### Statistical analysis

Statistical comparison between groups of mice was made using Fisher’s Exact Probability Test (incidence of the clonic seizure phase). The percentages of animals exhibiting clonic seizures were plotted against the corresponding doses by a computer construction of the dose- effect curves for calculation of CD_50_ (±95% confidence limits). The CD_50_ values for each compound were calculated using a computer program of the method of Litchfield and Wilcoxon, as previously described^30^. At least 32 animals were used to calculate each CD_50_ value. All other data are presented as means ± SEM. The statistical analyses were performed with the use of Graph‐Pad Prism (Graph‐Pad Software). For all the experimental data, we evaluated group differences with two‐way ANOVA followed by Tukey’s multiple comparison test. Statistical significance was set at p < 0.05.

## Results

### Evaluation of pentylentetrazol-induced seizure susceptibility

**Figure 2:**
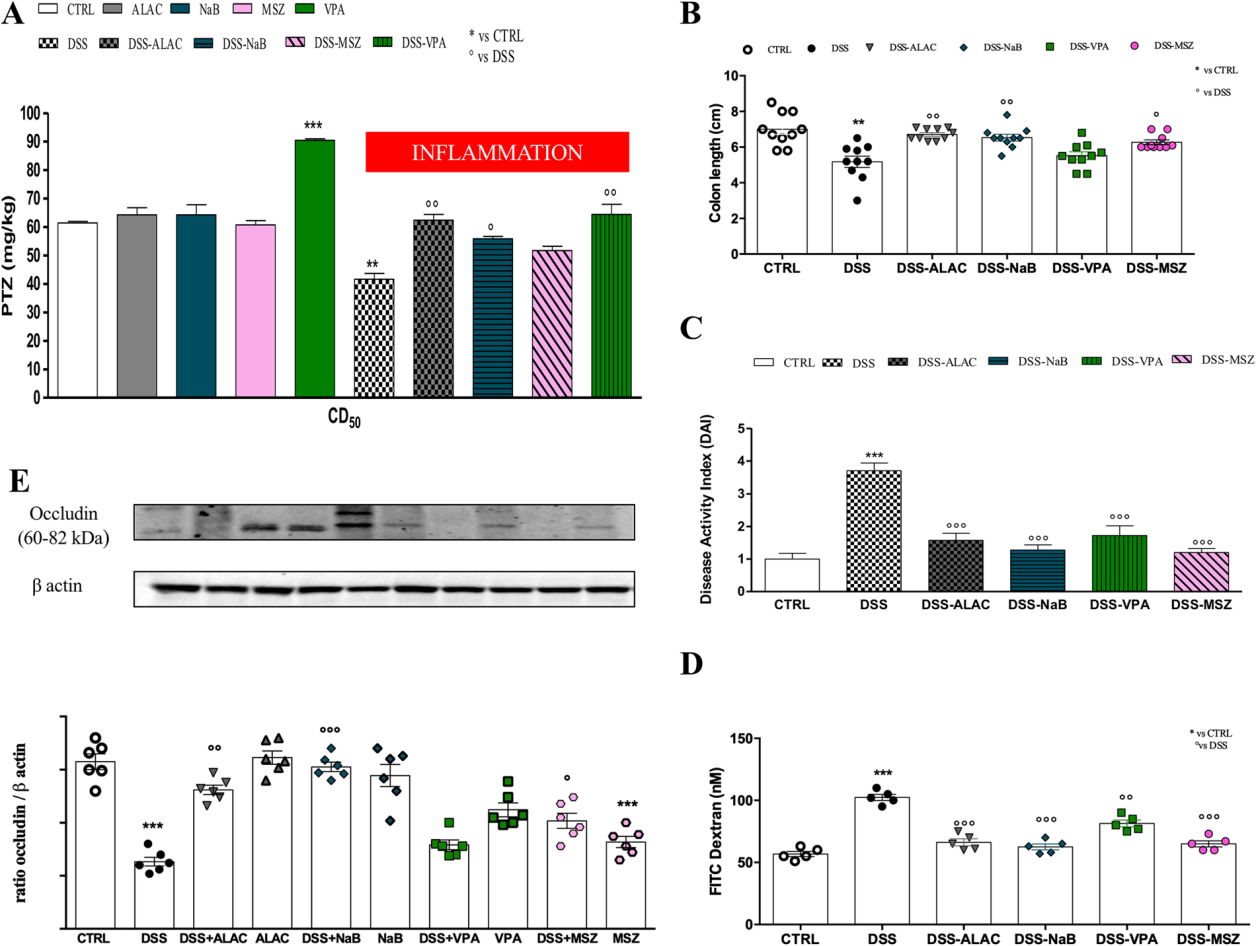
Treatment effects on. (**A**) PTZ-induced seizures measured by determination of CD_50_ (±95% confidence interval) in control and DSS (intestinal inflammation) mice groups (*n* = 28 for each group), VPA was the only effective drug in control mice while after induction of colitis, ALAC and NaB become effective; MSZ was not effective and VPA appeared less effective than in control mice; (**B**) colon length (*n* = 10 for each group) was shortened by DSS and recovered by all treatments with the exception of VPA. (**C**) Disease Activity Index; (*n* = 30 for each group) and gut permeability. (**D**) FITC Dextran; *n* = 5 for each group) were normalized by all treatments. (**E**) Occludin expression (*n* = 6 for each group), was normalized by ALAC and NaB treatments, significantly increased by MSZ but not modified by VPA. Data are means ± SEM and * indicates p < 0.05. ALAC = alpha-lactoalbumin; NaB = sodium butyrate; CTRL = control; DSS = dextran sulfate sodium; MSZ = mesalazine; PTZ = pentylenetetrazol; VPA = valproic acid.

Mice in all groups were treated with various doses of PTZ and CD_50_ values were calculated for each group and reported in Fig. 2A. In the control mice group, the calculated PTZ CD_50_ was 61.96 mg/kg and in the mice without DSS-induced colitis, only VPA (600 mg/kg) was able to significantly increase the CD_50_ (89.94 mg/kg) whereas the other treatments (ALAC, NaB and MSZ) were not effective. On the other hand, DSS-induced colitis significantly decreased the PTZ CD_50_ (43.68 mg/kg) relative to control non-DSS treated mice, indicating a decreased seizure threshold. Mesalazine treatment in these DSS-treated mice did not modify susceptibility to PTZ-induced seizures while, notably, the previously ineffective doses of ALAC (375 mg/kg) and NaB (100 mg/kg) were significantly effective in increasing the CD_50_ in comparison to the DSS-control group with ALAC, maintaining the CD_50_ to about the same value as that obtained in CTRL mice (not DSS treated) and with NaB only slightly lower. Finally, VPA was still effective in increasing PTZ CD_50_ in DSS-treated mice, however its effects were much lower in comparison to that obtained in non DSS-treated mice. The CD_50_ in this latter group was 89.94 mg/kg with an increase in comparison to control of 27.98 mg/kg and in the DSS-treated mice group this increase was of 24.29 mg/kg and therefore similar.

### Evaluation of experimental colitis and gut permeability

Drug treatments in all control groups did not affect the DAI (data not shown). However, all treatments in a similar fashion, reduced DAI showing significant protective effects against DSS-induced colitis (Fig. 2B). Similarly, DSS-dependent tissue shortening (Fig. 2C) was recovered by all treatments with the exception of VPA, and gut permeability as measured by FITC dextran was increased by DSS and recovered by all treatments (Fig. 2D). No differences were observed between drug-treated groups *vs* CTRL group.

Finally, occludin expression was significantly (p < 0.005; F_(1,50)_ = 8.30) reduced by DSS treatment and surprisingly also by MSZ treatment (p < 0.01; F_(4,50)_ = 3.78) in control animals. While in DSS-treated groups, ALAC and NaB treatments recovered occludin expression to control levels, MSZ significantly (p < 0.001; F_(4,50)_ = 7.79) increased it to about 75% of control and VPA, instead, had no effect (Fig. 2E).

### Histological analysis and scoring of colon sections

**Figure 3:**
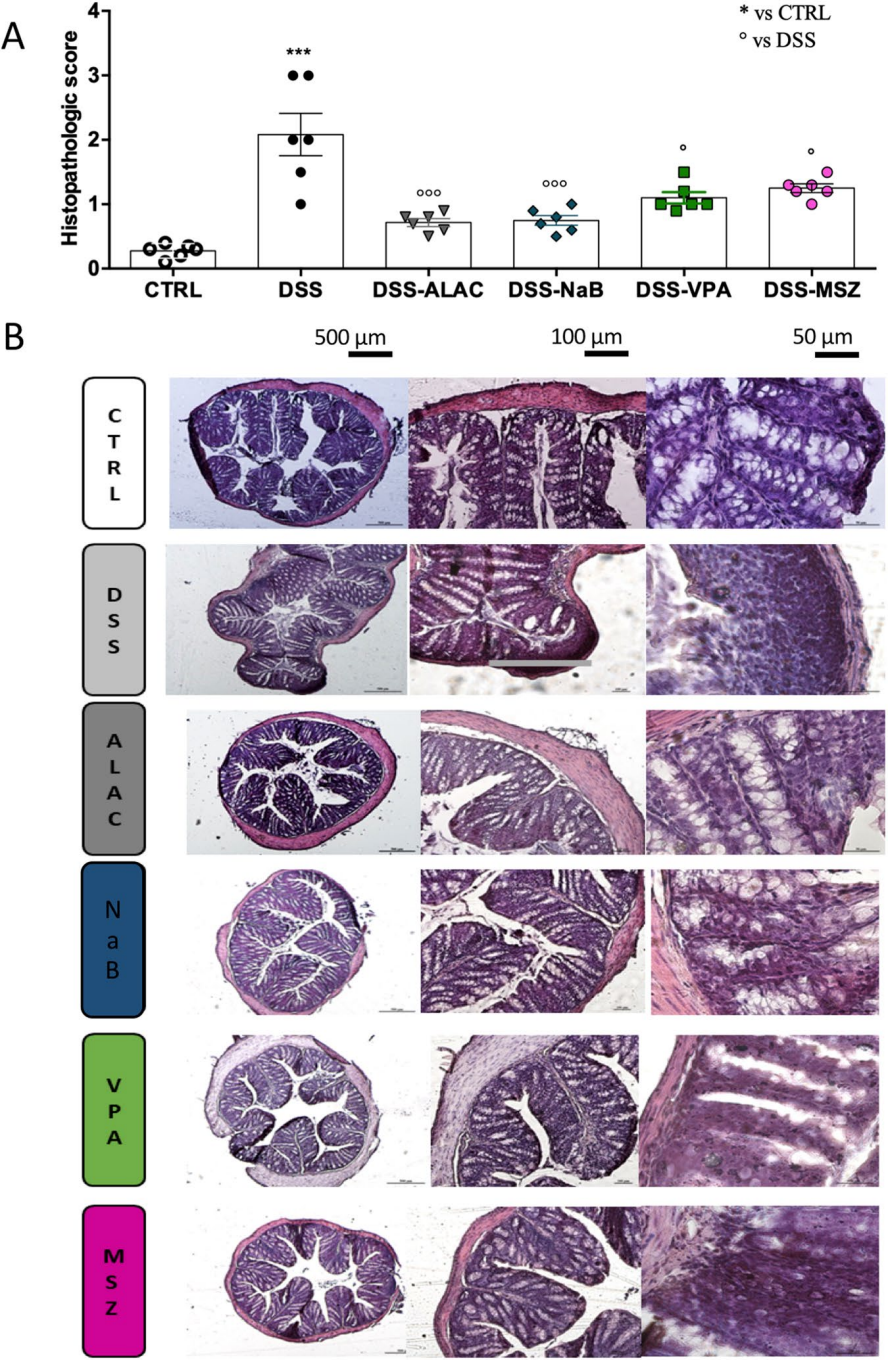
Drug effects on colon histology (*n* = 6 for each group). (**A**) All treatments were able to protect colonic mucosa structure, ameliorating the mucosal integrity and crypt structure and improving the epithelial surface compared with the DSS-treated group. (**B**) Control colon sections showed an intact epithelium, well‐defined crypt length, no edema in the mucosa and submucosa, and no ulcers or erosions. In contrast, colon tissue from the DSS group showed severe inflammatory lesions throughout the mucosa and loss of crypt architecture. Data are means ± SEM and * indicates p < 0.05. ALAC = alpha-lactoalbumin; Na Bu = sodium butyrate; CTRL = control; DSS = dextran sulfate sodium; MSZ = mesalazine; PTZ = pentylenetetrazol; VPA = valproic acid.

Control colon sections showed an intact epithelium, well‐defined crypt length, no edema in the mucosa and submucosa, and no ulcers or erosions (Fig. 3B). In contrast, colon tissue from the DSS-treated group showed severe inflammatory lesions throughout the mucosa and loss of crypt architecture. All treatments were able to protect colonic mucosa structure, ameliorating the mucosal integrity and crypt structure and improving the epithelial surface compared with the DSS group (Fig. 3B). The beneficial effects mediated by treatments were also confirmed by evaluation of the histopathological score of distal colon sections stained with H&E (Fig. 3A). On a scale, ALAC and NaB were the treatments with the highest protecting score (both about 0.8), followed by VPA and MSZ (both about 1.2).

### Western blot analysis for inflammatory parameters

**Figure 4:**
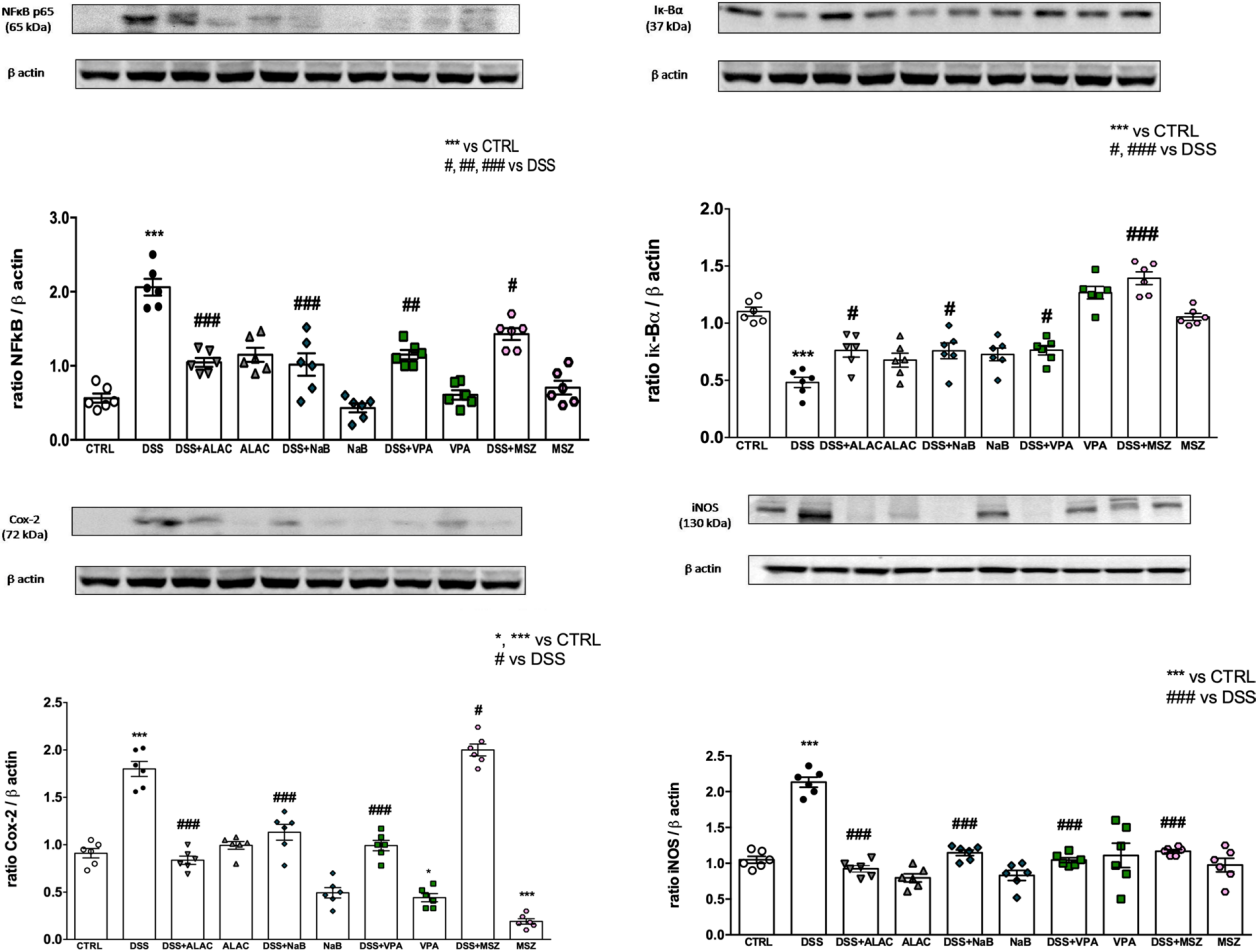
Western blot analysis of inflammatory parameters (*n* = 6 for each group). (**A**) NF-κB; (**B**) Iκ-Bα; (**C**) COX-2 and (**D**) iNOS. All drugs are able to rescue DSS inflammatory effects. Data are means ± SEM and *indicates p < 0.05. ALAC = alpha-lactoalbumin; NaB = sodium butyrate; COX-2 = cyclooxygenase type 2; CTRL = control; DSS = dextran sulfate sodium; iNOS = inducible nitric oxide synthase; MSZ = mesalazine; PTZ = pentylenetetrazol; VPA = valproic acid.

Protein concentrations of colon lysate were evaluated with specific antibodies in order to study the effects of DSS and drug treatments on inflammatory proteins. NF-κB expression was increased 4-fold by DSS-treatment (p < 0.001; F_(1,50)_ = 11.57) and accordingly Iκ-Bα was decreased 2-fold (P < 0.003; F_(1,50)_ = 9.83) while drug treatments in non-DSS-treated animals had no effects on NF-κB (Fig. 4A). At odds with this, Iκ-Bα expression was significantly (p < 0.004 F_(4,50)_ = 4.26) reduced by ALAC and NaB treatments in CTRL animals while VPA and MSZ treatment had no effects in this experimental group. On the other hand, in DSS-treated mice, MSZ was not effective while all other treatments were able to counteract the induced increase of NF-κB. In this experimental group, Iκ-Bα expression was normalized by all treatments and MSZ, in this case, was the most effective on this parameter, being significantly different in comparison to all other DSS-treated groups and even significantly higher than CTRL group (Fig. 4B).

COX-2 expression was ~2-fold increased by DSS treatment (p < 0.002; F_(1,50)_ = 9.87), and notably significantly reduced by VPA and MSZ treatment in non-DSS-treated mice. However, ALAC, NaB and VPA were able to significantly normalize DSS effects (p < 0.007; F_(4,50)_ = 4.05) while, surprisingly, MSZ was not effective in this case (Fig. 4C).

iNOS concentration was ~2-fold increased by DSS-treatment (p < 0.006; F_(1,50)_ = 7.98) while drug treatments did not alter its expression in control animals while completely preventing its increase in DSS-treated groups with no clear differences between drugs (Fig. 4D).

### GC-MS propionate and butyrate quantification

**Figure 5:**
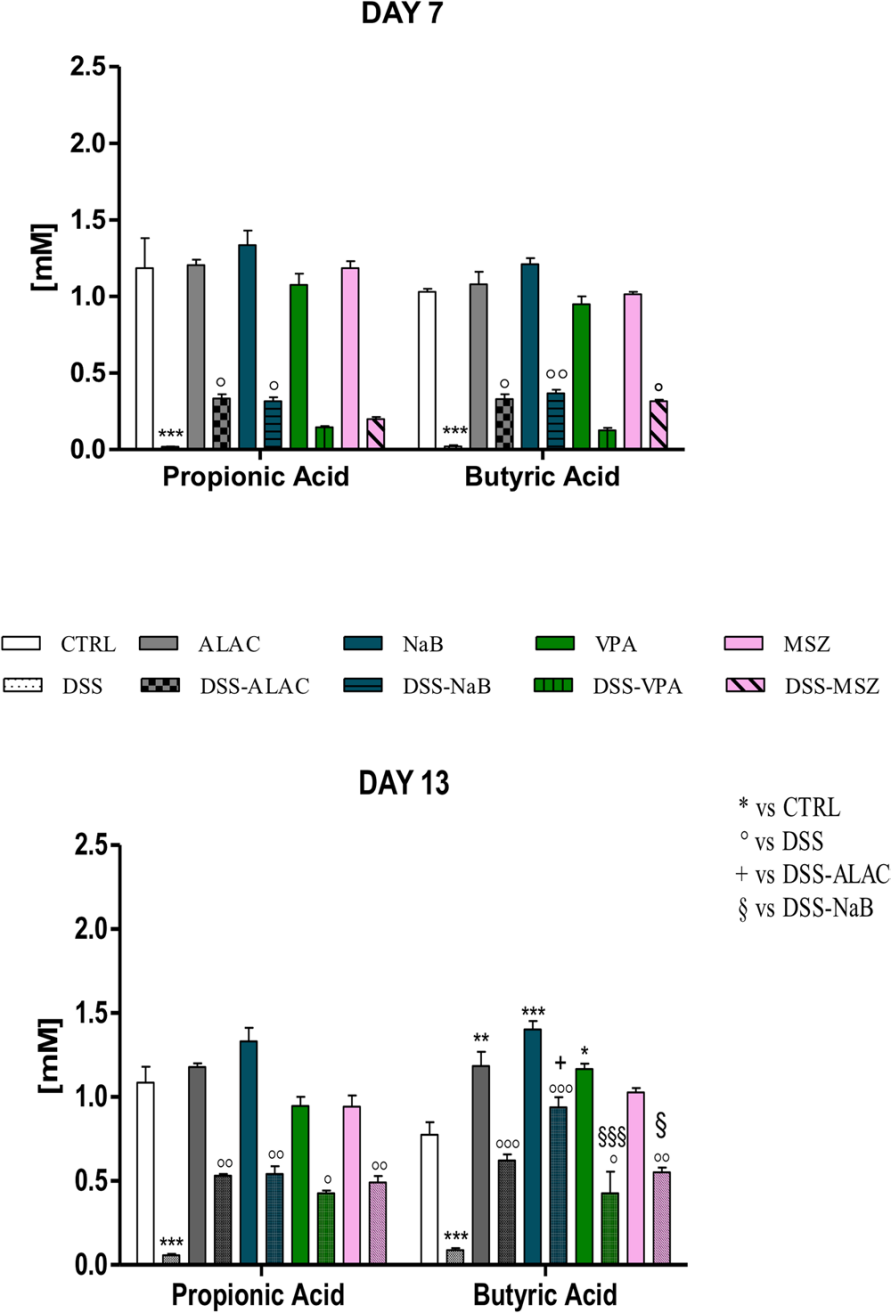
Drug effects (*n* = 6 for each group) on propionic and butyric acids levels in stools both at day 7 and 14 after initiation of DSS treatment which, *per se*, nearly completely abolished production of both acids by the gut-microbiota (dysbiosis) and treatments differently affected acid levels, generally increasing their amounts. Data are means ± SEM and *indicates p < 0.05. ALAC = alpha-lactoalbumin; NaB = sodium butyrate; CTRL = control; DSS = dextran sulfate sodium; MSZ = mesalazine; PTZ = pentylenetetrazol; VPA = valproic acid.

Propionate and butyrate concentrations in feces were measured both at experimental day 7 (1 day after the end of DSS administration) and on experimental day 13 (before mice sacrifice and sampling, see Fig. 5). DSS treatment, in both cases, virtually abolished (p < 0.008; F_(1,50)_ = 56) the production of these SCFAs in the intestine which were nearly undetectable in stool samples. None of the treatments modified propionate and butyrate concentrations in control animals at day 7 (after 4 days) while at day 13 (at 10 days of treatment) butyric acid concentration was significantly increased by all treatments with the exception of MSZ (Fig. 5). In DSS-treated experimental groups, all drugs were effective at rescuing SCFA production, this effect was already significant at experimental day 7 (with VPA exception) and on experimental day 14, while propionate was still about half the amount of the control group for all drugs, and butyrate levels were not significantly different from control. Significant effects were observed between drug groups; it can be noted that NaB treatment was significantly (p < 0.001; F_(4,50)_ = 5.17) more effective than VPA and MSZ (Fig. 5B).

## Discussion

The role of intestinal inflammation in reducing seizure threshold or aggravating seizures has been previously reported^7,15^. In this paper, we confirmed the link between gut inflammation and epilepsy in a different colitis animal model (*i.e*. DSS model). In addition, we demonstrated that drugs able to recover intestinal inflammation indeed had an effect on seizure threshold, whereas intestinal inflammation may reduce the efficacy of previously effective drugs. In particular, we showed that the reduction of intestinal inflammation through ALAC treatment as well as Na butyrate treatment resulted in a strong antiseizure effect. Furthermore, our data support the view that intestinal inflammation may reduce the antiepileptic effects of VPA and confirmed that it lowers seizure threshold.

The mechanisms by which intestinal inflammation can lead to a higher seizure susceptibility has not yet been completely clarified. It has been previously demonstrated that induction of colitis by 2,4,6-trinitrobenzene sulfonic acid (TNBS) increases cytokine levels (IL-1β and TNF-α) in the hippocampus and that TNF-α is the most important in mediating microglia activation and this was linked to a higher seizure susceptibility^15^. In our study, all treatments were in fact able to reduce intestinal damage and inflammation and an unclear proportionality can be defined within this set of experiments; indeed, a dose-response analysis and an in-depth evaluation of several parameters could try to define the exact mechanisms involved. However, these results can be interpreted with a specific action of ALAC and NaB in counteracting the proconvulsant role of intestinal inflammation which could be mediated by several agents spanning from the protective role to the involvement of other factors. For example, ALAC may exert its effect by an increase in tryptophan levels and its metabolites such as kynurenic acid which may penetrate the brain and act on N-methyl-D-aspartate (NMDA)-type glutamate receptors, as previously suggested^17,18^. Similarly, NaB may have also acted directly on the brain, considering its ability to modulate several processes^28^. In contrast, MSZ was not effective on seizure threshold while it was able to protect the intestine, similarly to ALAC and NaB, indicating that removing inflammation *per se* might not necessarily be enough to obtain an effect on the CNS. On the other hand, VPA effects were notably dampened by colitis even if the drug was also able to reduce intestinal inflammation; this latter anti-inflammatory effect by VPA was already reported both on the nervous system^32,33^ and peripheral organs^34^. The apparently reduced VPA efficacy may have several explanations; for example, VPA absorption may have been reduced by intestinal inflammation and therefore its final brain concentration may have been lower. Nevertheless, it is particularly relevant that in the case of an intestinal inflammation, as could happen in clinical practice, the antiepileptic effect of VPA could be reduced and this may lead to the appearance or aggravation of uncontrolled seizures in patients with epilepsy.

Recently, the gut-brain axis and the role of microbiota in brain diseases including epilepsy has gathered attention with mounting evidence of a strict connection; for example, it is known that chronic restraint stress or social defeat stress are able to trigger dysbiosis along with a possible involvement of the gut-microbiota-brain axis^35–37^ and microbiota alteration due to stress was involved in the facilitation of kindling in rats^38^. It was then suggested that dysbiosis and/or microbiome reshaping may be relevant in several areas of epilepsy research including traumatic brain injury or biomarkers. Furthermore, the well-recognized antiepileptic ketogenic diet was convincingly demonstrated to have a modulating effect on gut microbiota with this latter aspect being necessary for its action^39,40^. DSS-induced colitis has as a consequence, an alteration of gut microbiota (*i.e*. dysbiosis) which is also confirmed in our experiments indirectly by the strong reduction in the amount of propionic and butyric acids in the stools^41^.

In our study, ALAC and NaB better restored SCFA levels and were the most effective drugs on seizure threshold in the PTZ mouse model although it remains to be determined what the contribution of microbiota were to increased seizure susceptibility. In fact, ALAC and NaB were both more effective compared with VPA and MSZ on a number of parameters in reducing intestinal inflammation, probably with an impact on microbiota and on circulating inflammatory mediators linked to peripheral inflammation. Therefore, the CNS effects observed may be linked to more than one single parameter. It is intriguing, however, to believe that seizures may arise from an inflamed intestine and that drugs acting on this inflammation may prove to be effective antiepileptics, even if the lack of MSZ effects suggest that a general intestinal anti-inflammatory action is not sufficient, but that a more specific intervention leading to probable restoration of a healthy intestine is more effective.

In conclusion, our data suggest that in patients with epilepsy, the intestine and its physiopathological state may also contribute to the disease and in some cases an appropriate drug intervention aimed at restoring a “*normal*” gut function may have beneficial anticonvulsant effects. In addition, it has to be underlined that an alteration of intestinal function can reduce drug effects and that a specific clinical evaluation should be always performed. Finally, it will be crucial to further understand whether other mediators than known inflammatory factors are involved in the link between inflammation and epilepsy and studying the role of gut microbiota is currently one of the most promising research areas confirming its relevance as a potential therapeutic target for this condition.

## Supplementary information

Suppmenetary data set 1

## Data Availability

Data may be available upon request.
