# Supplementary material for: Intestinal inflammation increases convulsant activity and reduces antiepileptic drug efficacy in a mouse model of epilepsy
Source: Sci Rep. 2019 Sep 27;9:13983. doi: 10.1038/s41598-019-50542-0 (PMC6764994; doi:10.1038/s41598-019-50542-0)

Intestinal inflammation increases convulsant activity and reduces antiepileptic drug efficacy in a mouse model of epilepsy

Carmen De Caro, Ph.D.[1], Antonio Leo, Ph.D.[1], Valentina Nesci, Ph.D.[1], Carla Ghelardini, Ph.D.[2], Lorenzo di Cesare Mannelli, Ph.D.[2], Pasquale Striano, MD[3], Carmen Avagliano, Ph.D.[4], Antonio Calignano, Ph.D.[4], Paolo Mainardi[5], Andrew Constanti, Ph.D.[6], Rita Citraro, Ph.D.[1], Giovambattista De Sarro, MD[1], Emilio Russo, MD, Ph.D.[1,*]

iNOS

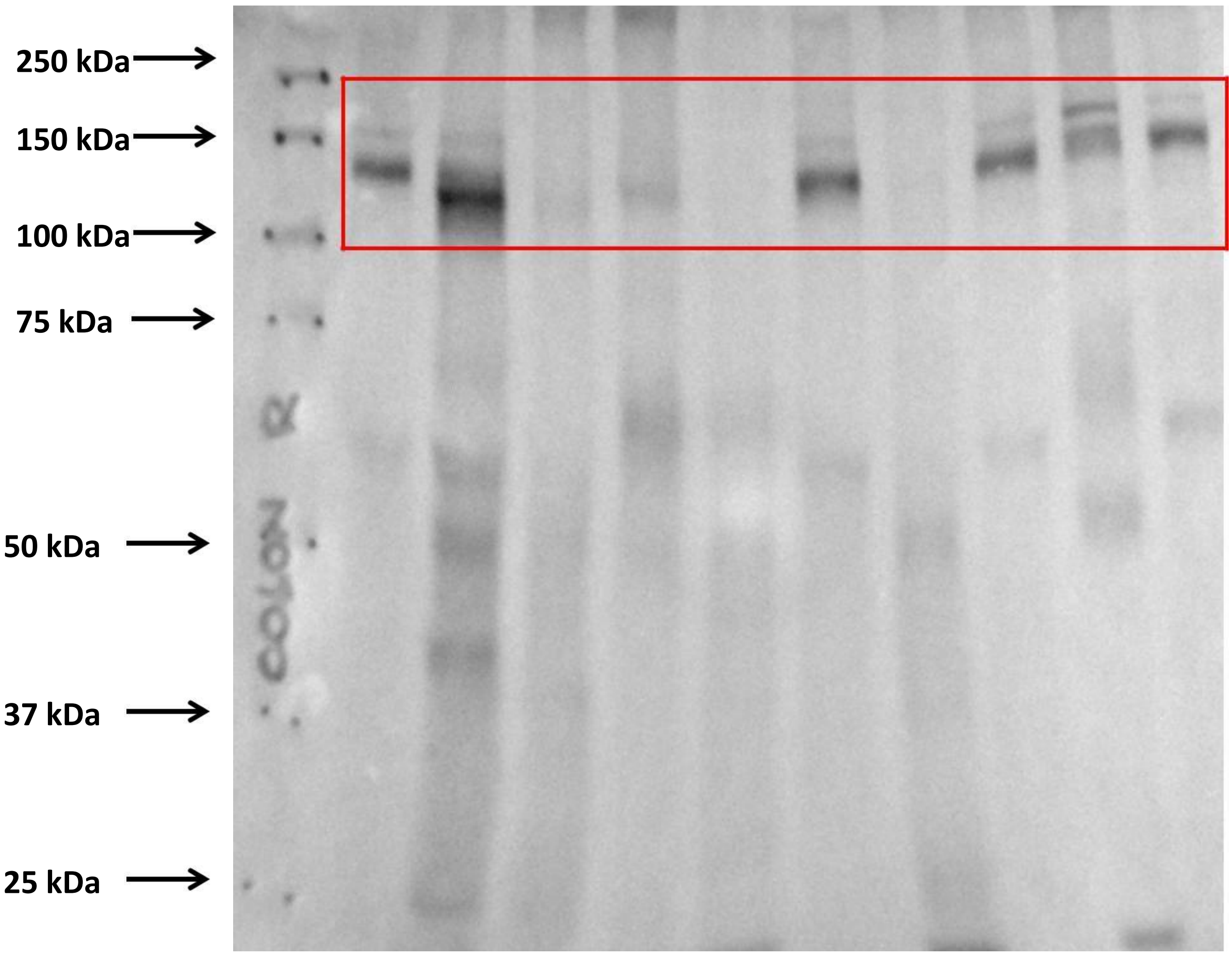

NFkB

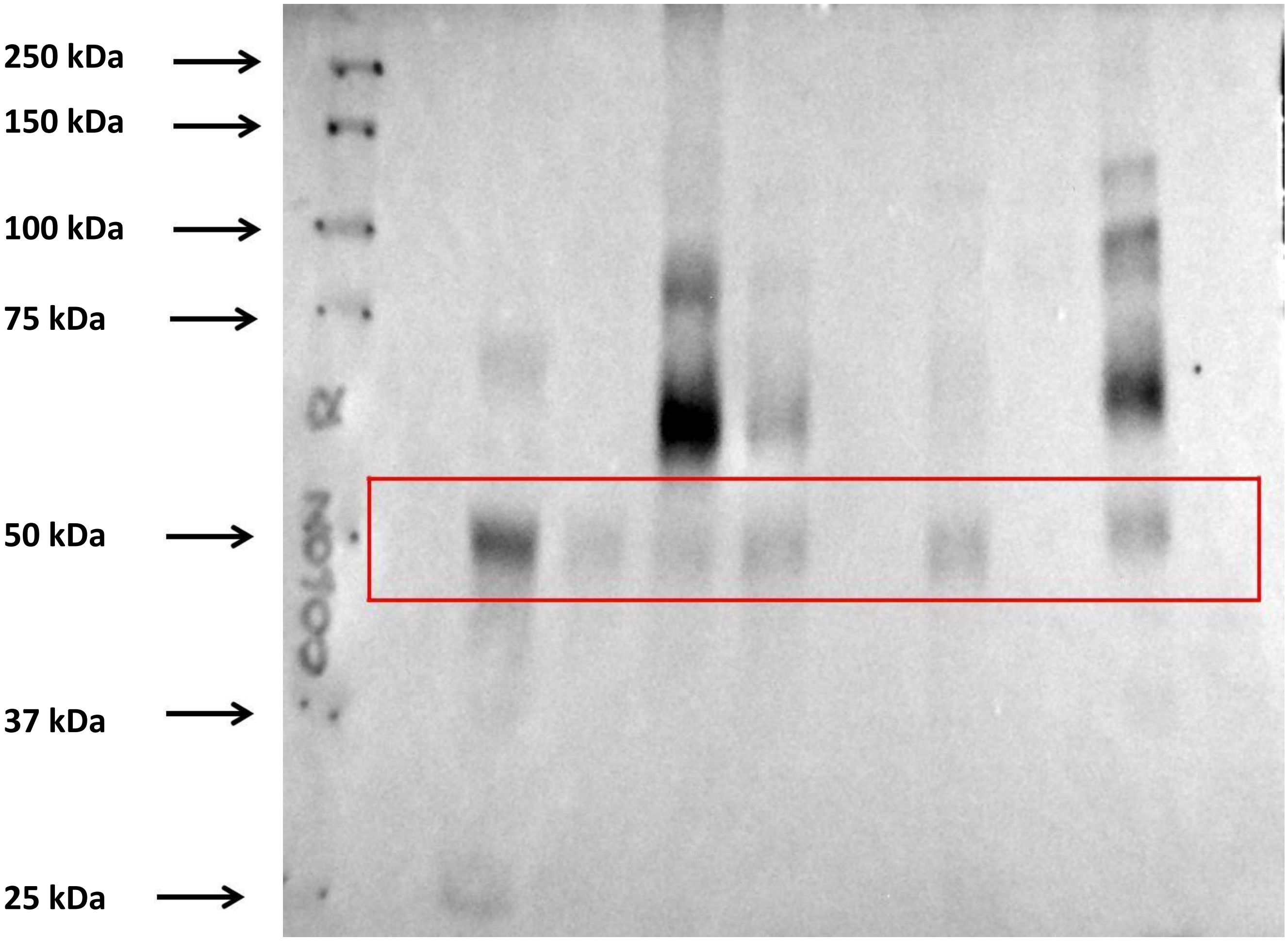

COX-2

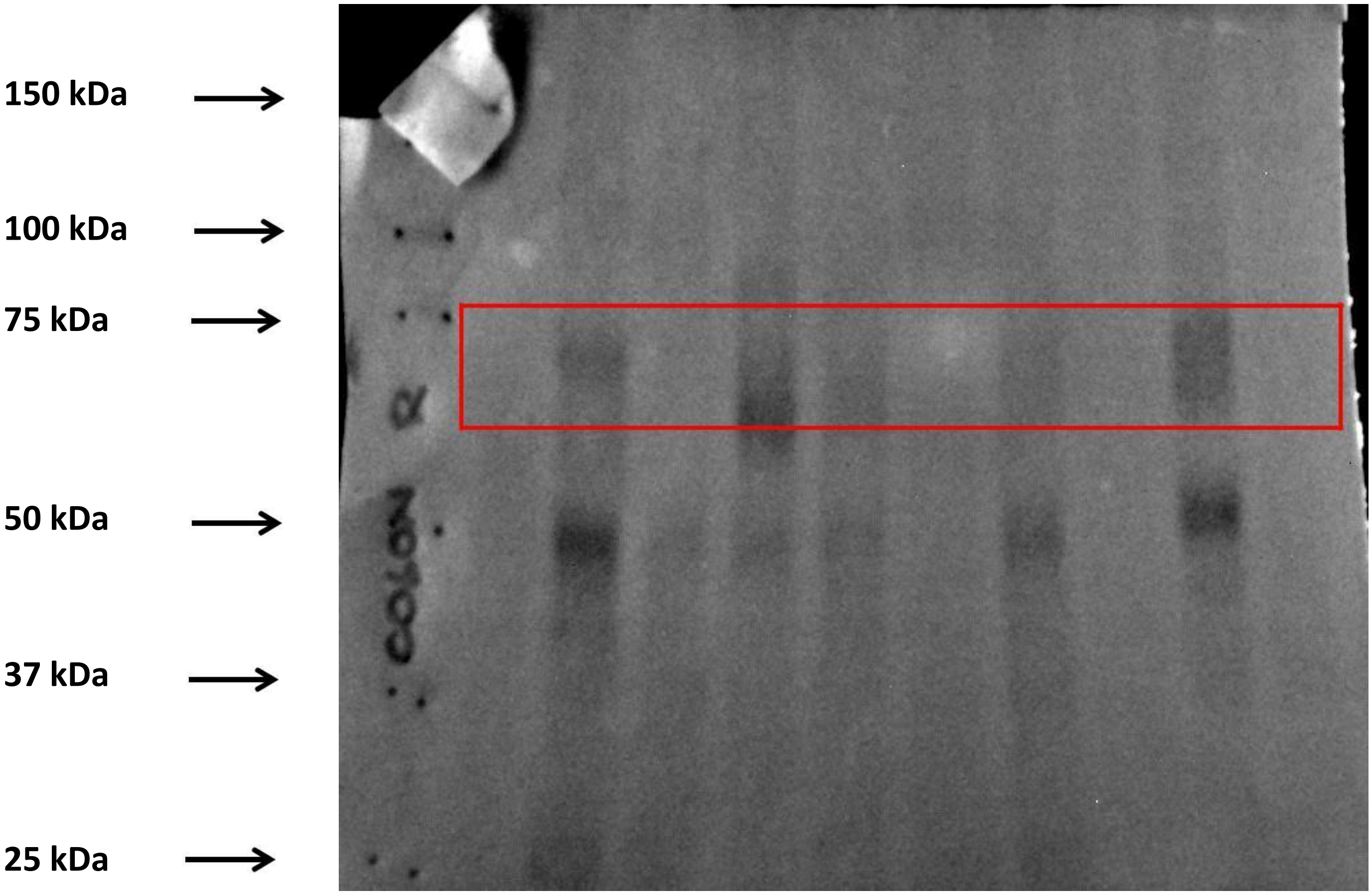

ikB alpha

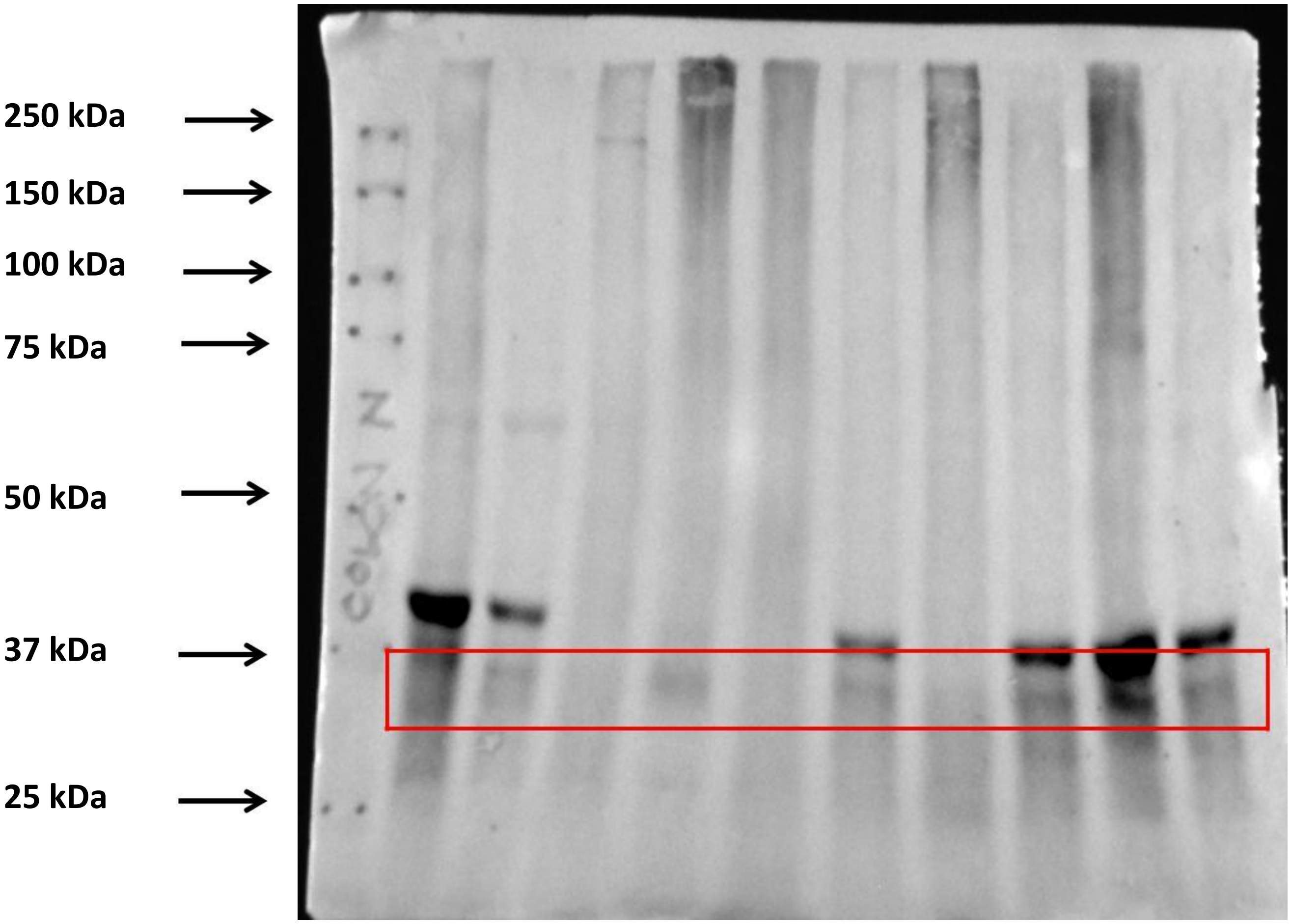

occludin

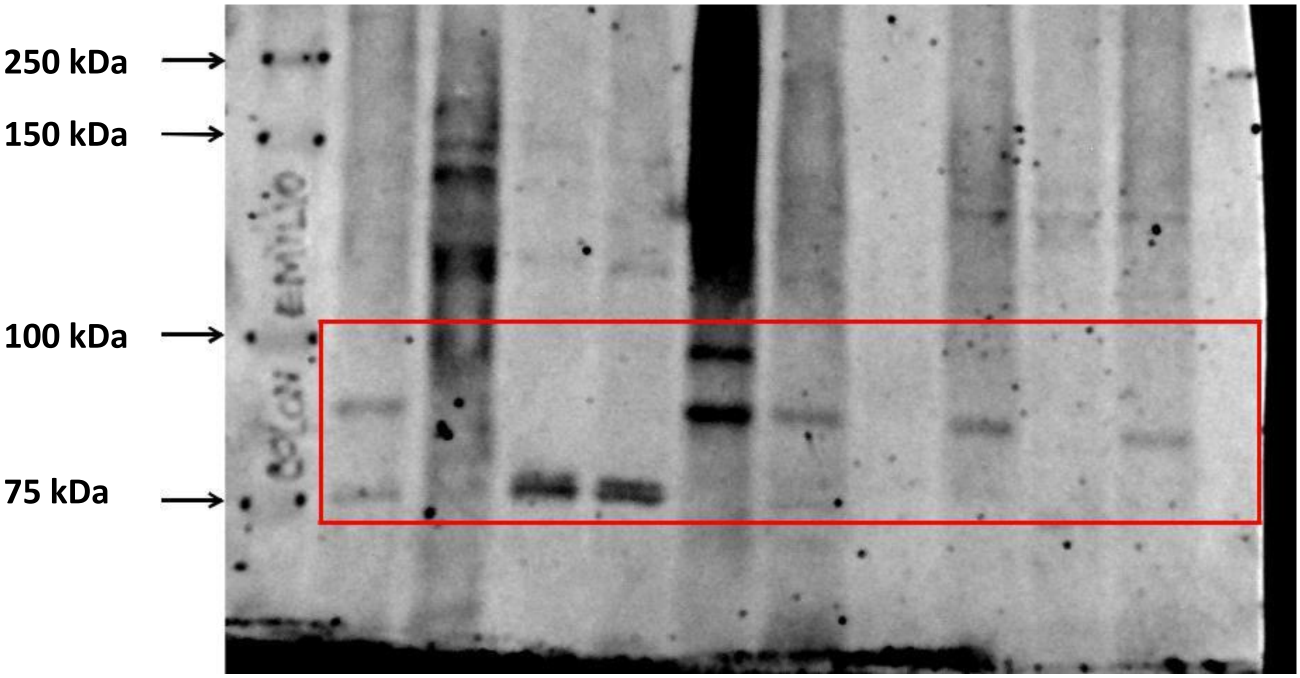

β actin

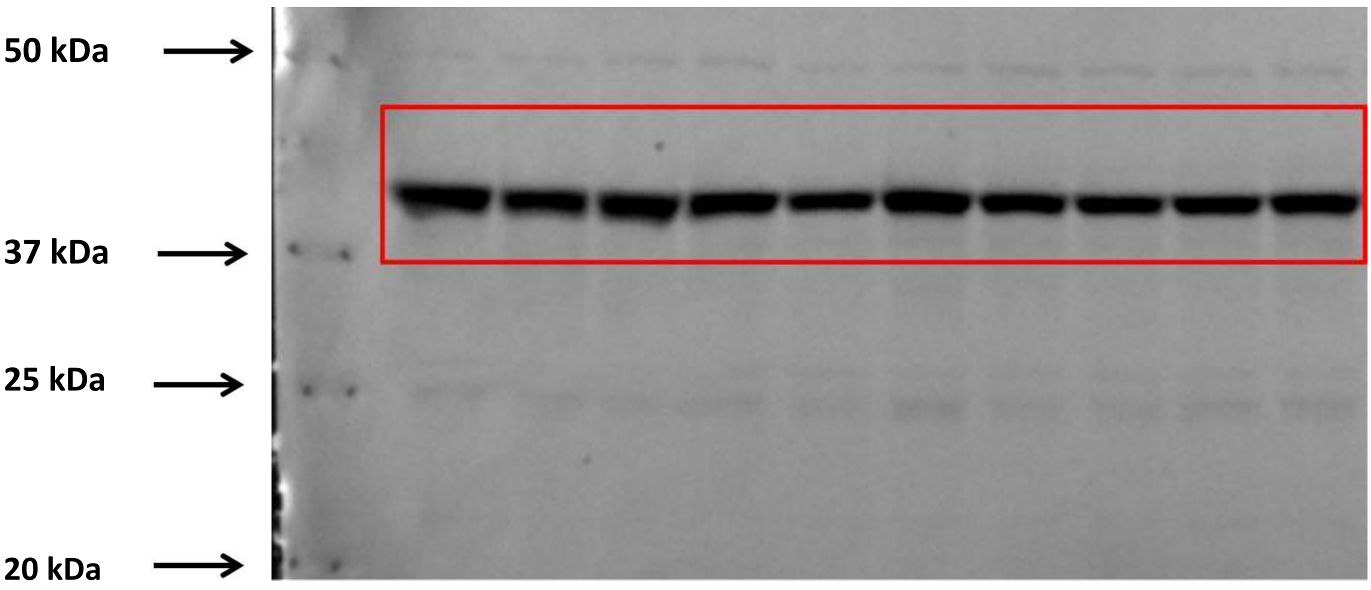

iNOS

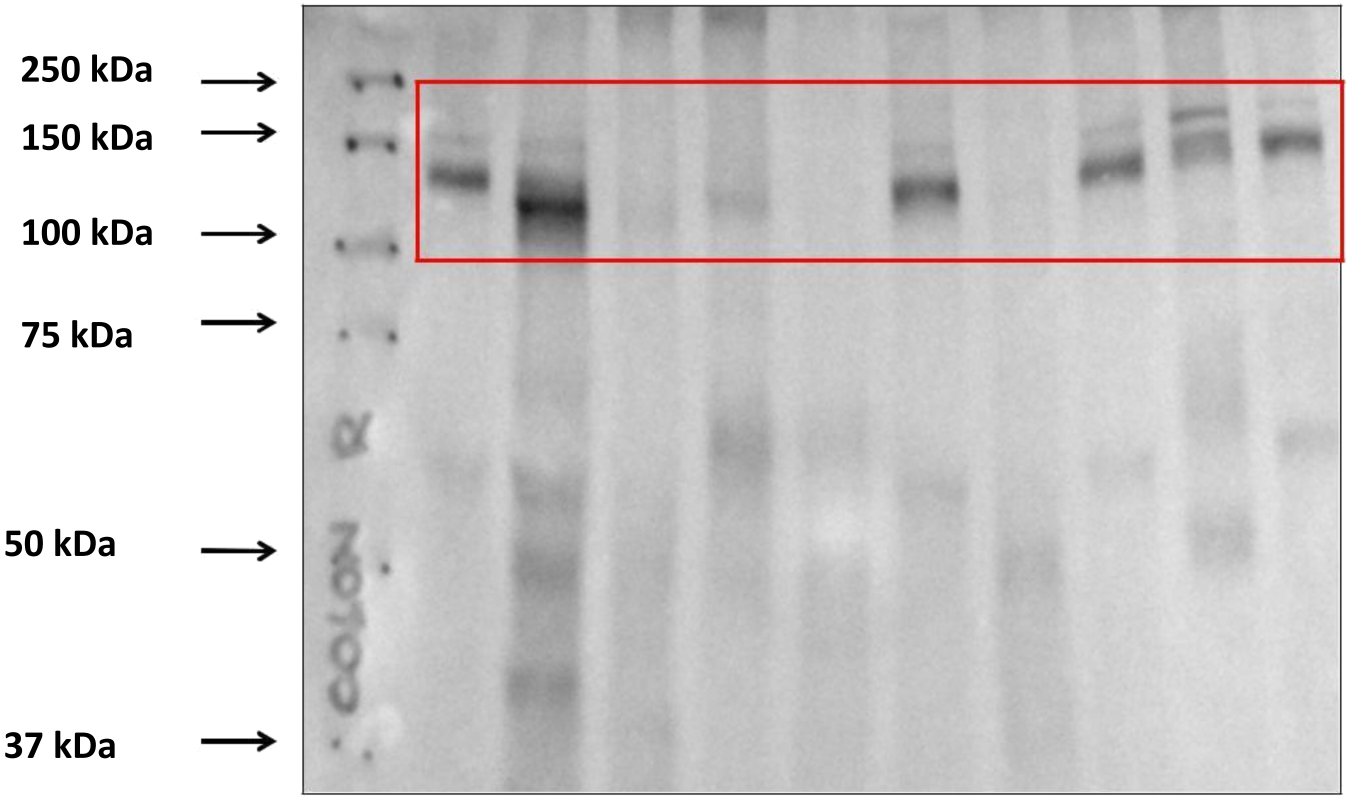

NFkB

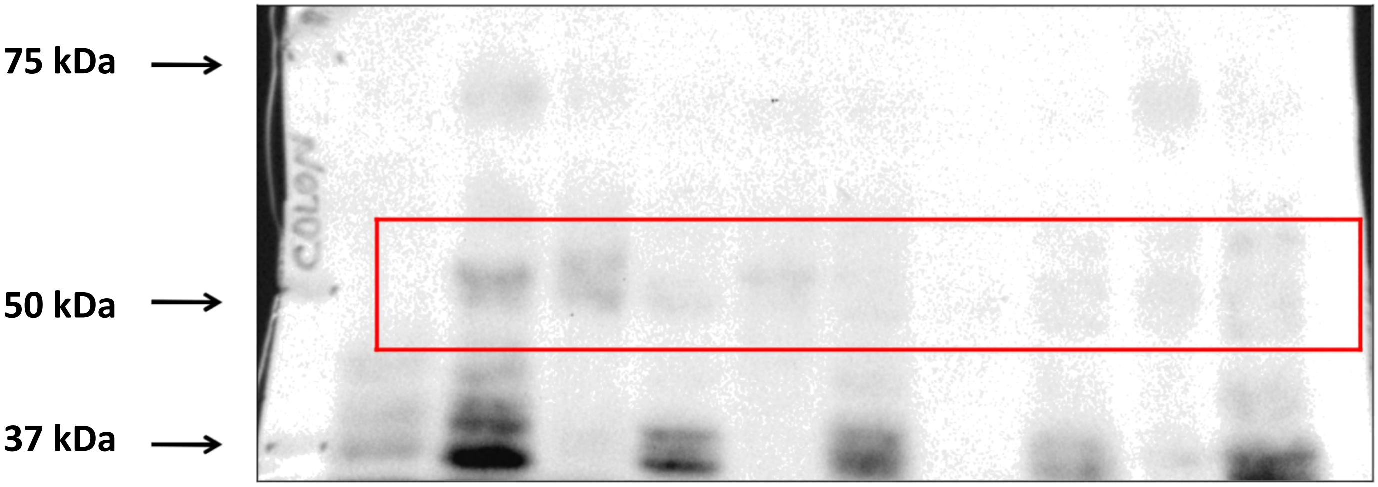

COX-2

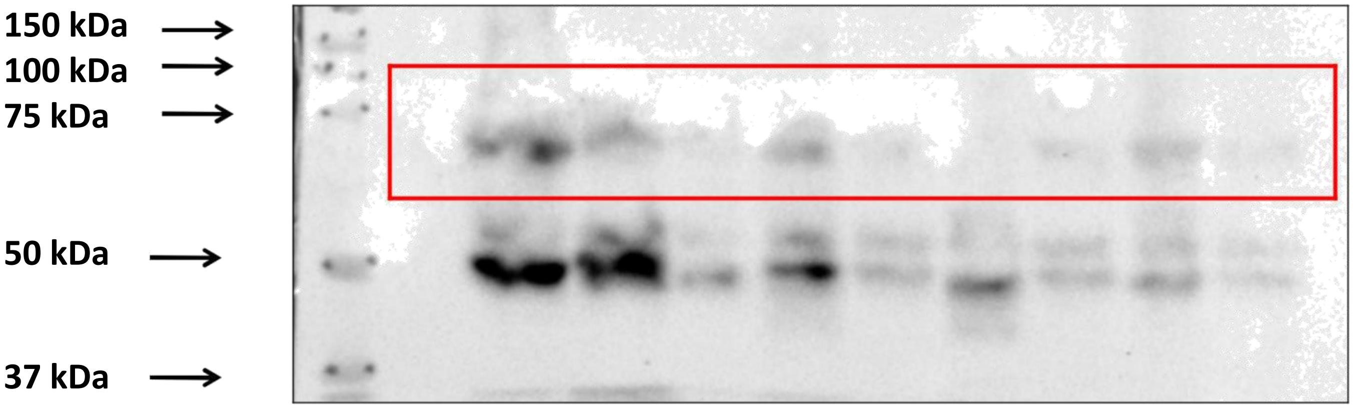

ikB alpha

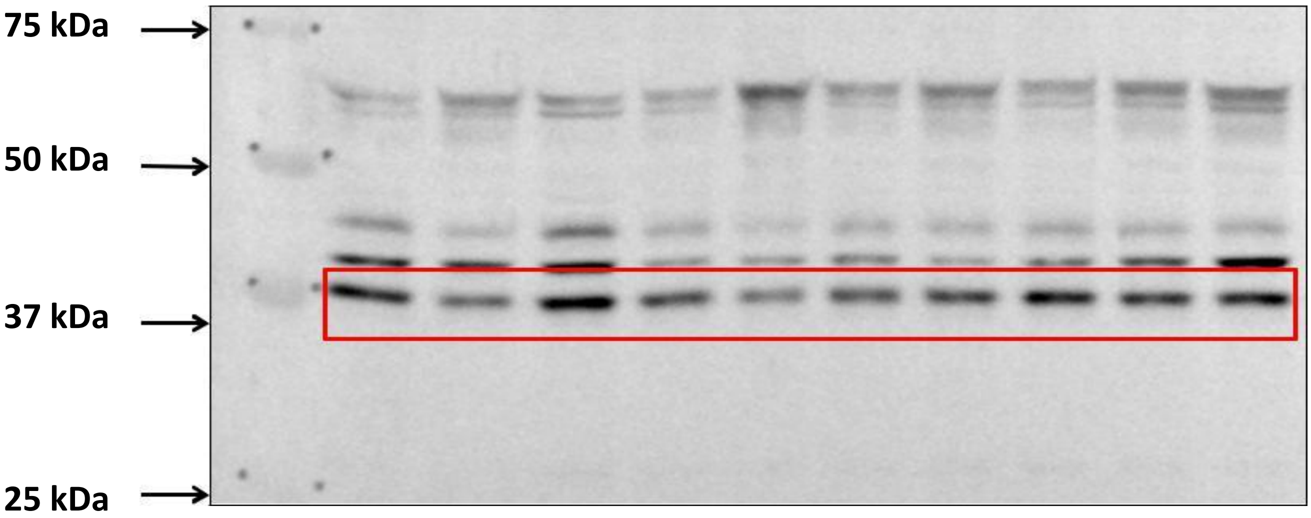

occludin

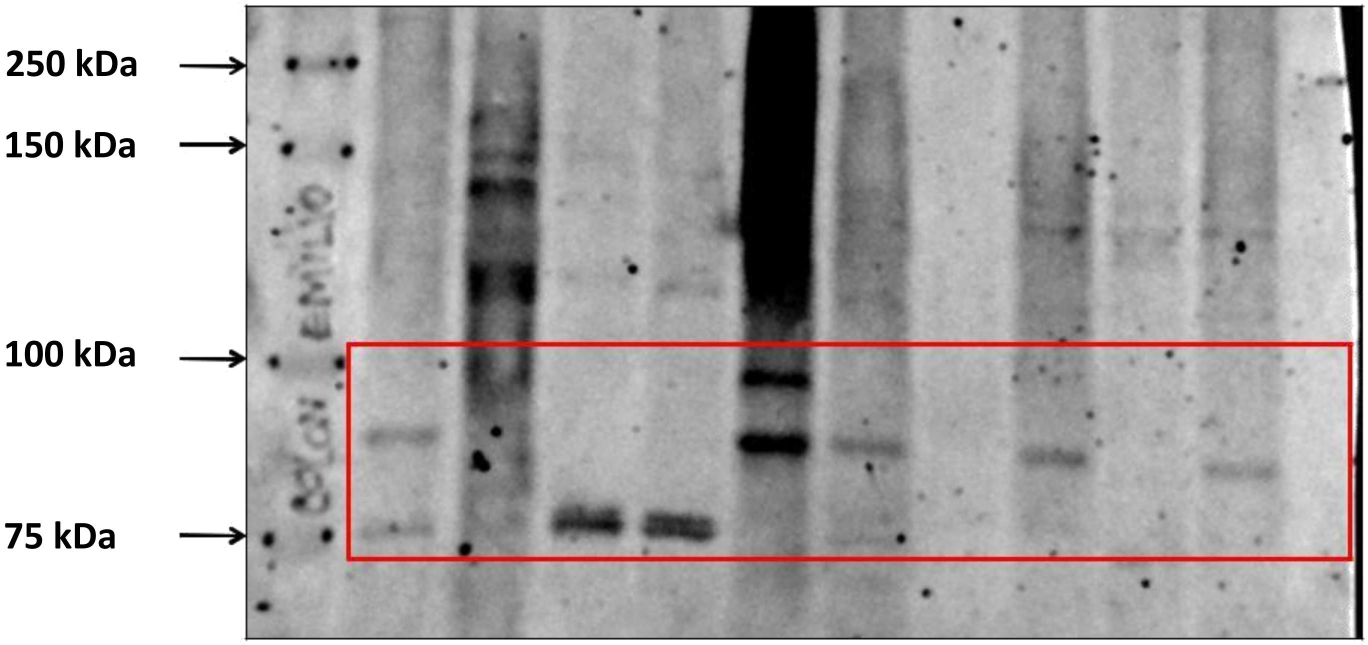

β actin

50 kDa →

37 kDa →

25 kDa →

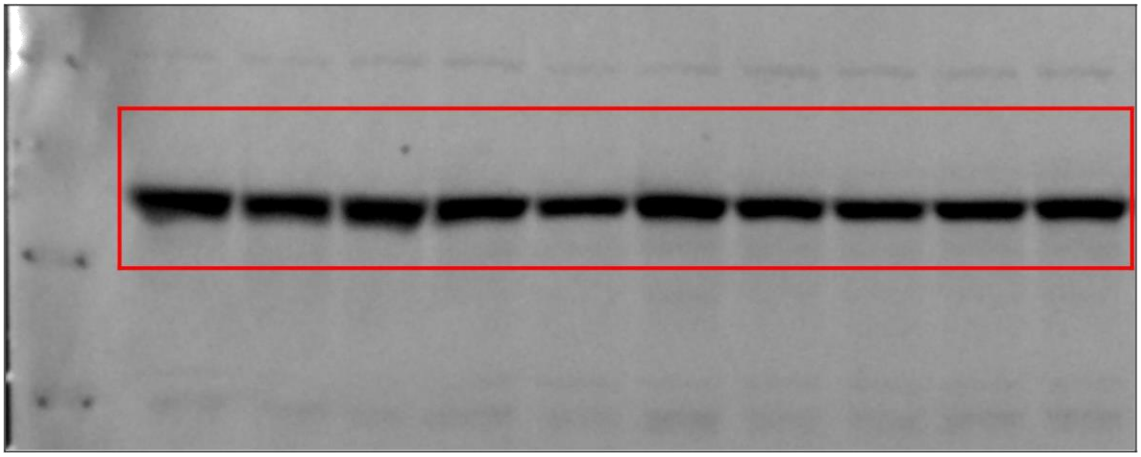

β actin

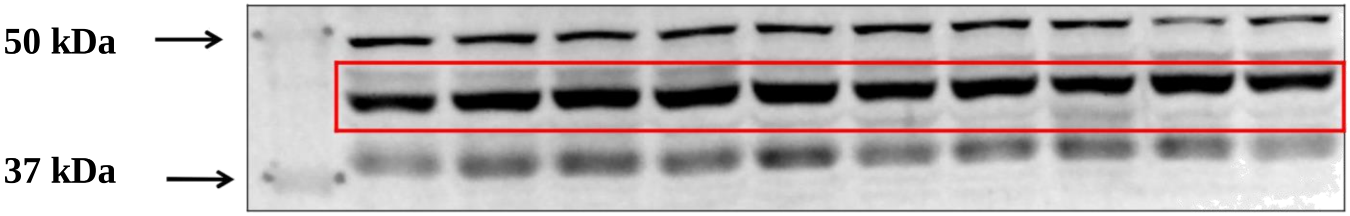

Supplement: Supplementary file 1 — Suppmenetary data set 1 [file 41598_2019_50542_MOESM1_ESM.pdf]
